# Peripheral muscle oxygenation during upper extremity functional exercise and balance in pediatric patients with cystic fibrosis: a cross-sectional study

**DOI:** 10.1007/s00421-026-06130-y

**Published:** 2026-03-07

**Authors:** Betül Yoleri, Meral Boşnak Güçlü, Tuğba Şişmanlar Eyüboğlu, Ayşe Tana Aslan

**Affiliations:** 1 Faculty of Health Sciences, Department of Cardiopulmonary Physiotherapy and Rehabilitation, Gazi University, Emek, Ankara, 06490 Turkey; 2 Institute of Health Sciences, Department of Physiotherapy and Rehabilitation, Gazi University, Ankara, Turkey; 3 Faculty of Medicine, Department of Pediatric Pulmonology, Gazi University, Ankara, Turkey

**Keywords:** Cystic fibrosis, Exercise tolerance, Near-infrared spectroscopy, Postural balance, Muscle strength

## Abstract

**Background/aim:**

Exercise intolerance, muscle weakness, mitochondrial involvement, and slow phosphocreatine metabolism are common, but extrapulmonary impairments in pediatric patients with cystic fibrosis (CF) have not been adequately studied yet. This study compared pulmonary function, upper extremity exercise capacity, muscle oxygenation, peripheral muscle strength, and balance in patients with CF and healthy controls.

**Materials and methods:**

Thirty-one pediatric patients with cystic fibrosis and 30 healthy controls were compared. Pulmonary function was assessed by spirometry; upper extremity functional exercise capacity was evaluated using the 6-minute Pegboard and Ring Test (6PBRT); deltoid muscle oxygenation was measured at rest, during the 6PBRT, and during recovery using near-infrared spectroscopy; shoulder abductor and knee extensor isometric muscle strength were assessed using a hand-held dynamometer; and static and dynamic balance were evaluated using the Biodex Biosway^®^ Portable Balance System and the Y-Balance Test, respectively.

**Results:**

Age and gender were similar in groups (*p* > 0.05). FEV_1_%, FVC%, PEF%, FEF_25 − 75_%, and muscle strength statistically significantly (*p* < 0.05) decreased in patients. Six-minute PBRT score, deltoid muscle oxygen saturation, and hemoglobin level were similar in groups (*p* > 0.05). The medial-lateral stability index of patients on soft ground with eyes open was statistically significantly higher than healthy controls (*p* < 0.05).

**Conclusion:**

Upper extremity exercise capacity, muscle oxygenation, and most parameters of balance are preserved, and peripheral muscles are weakened in mildly impaired patients with CF. Impaired pulmonary function may impair upper extremity exercise capacity and muscle oxygen metabolism in severe patients with CF, should be investigated.

## Introduction

Increased airway resistance, air trapping, and decreased flow rates lead to reduced lung capacities and can cause ventilatory failure in patients with cystic fibrosis (CF). These respiratory issues, along with ventilation-perfusion mismatch and intrapulmonary arteriovenous shunting, limit aerobic capacity (Harun et al. 2016; Werkman et al. 2016). Additionally, factors such as cardiac involvement, mitochondrial dysfunction, altered muscle metabolism, peripheral muscle abnormalities, reduced muscle strength, physical deconditioning, low body mass, compromised nutritional status, and a sedentary lifestyle further decrease exercise capacity in these patients (Janssens et al. 2013). Besides peripheral muscle weakness, patients with CF may also experience mitochondrial damage, slow phosphocreatine metabolism, problems with molecule regeneration and store replenishment, abnormalities in action potential generation, a decrease in resting ATP levels, and impaired ATP production during exercise. Restrictions on the trunk’s ability to contribute to postural stability, due to increased resting respiratory load, diaphragm weakness, peripheral muscle weakness, and systemic inflammation, may lead to balance issues in patients with CF (Beauchamp et al. 2012; Smith et al. 2016; Tudorache et al. 2015). Based on this rationale, the present study hypothesizes that upper extremity exercise capacity, muscle oxygen metabolism, peripheral muscle strength, and static and dynamic balance are impaired in pediatric patients with CF.

Limited studies have investigated muscle oxygenation, peripheral muscle strength, and balance in patients with CF. Moreover, upper extremity functional exercise capacity has not yet been investigated. The primary aim of this study was to compare upper extremity exercise capacity and muscle oxygenation in pediatric patients with CF and healthy controls. The secondary aim was to compare peripheral muscle strength and balance between the two groups.

## Materials and methods

### Study design

This cross-sectional study was carried out in the cardiopulmonary unit of Gazi University Faculty of Health Sciences, Department of Physiotherapy and Rehabilitation. Pediatric patients were referred for pulmonary rehabilitation by the Gazi University Faculty of Medicine, Department of Pediatric Chest Diseases. Pulmonary function, peripheral muscle strength, and static and dynamic postural stability were assessed on the first day. Upper extremity exercise capacity and muscle oxygenation in the patients and healthy children were evaluated on the second day by an experienced physiotherapist who is an expert in the field. The primary outcomes are upper extremity muscle oxygenation and balance. The study received approval from the Gazi University Ethics Committee (No: 839 /07.12.2020) (Trial Registration Number: NCT04803643) and was conducted following the Declaration of Helsinki. Written informed consent was obtained from all pediatric patients with CF and healthy children, as well as from the parents of all participating children.

### Participants

Pediatric patients with CF who were routinely followed up by pediatric pulmonologists and referred for pulmonary rehabilitation between April 2021 and September 2022 were included in this study. The inclusion criteria for patients were a diagnosis of CF (Farrell et al. 2008), aged between 6 and 18 years, and being clinically stable. The exclusion criteria included diagnosed visual, hearing, vestibular, or neurological problems that could affect balance, a history of hospitalization within the previous month, participation in a planned aerobic exercise training program in the last three months, a history of COVID-19, smoking, orthopedic issues that might impact walking, a history of acute pulmonary exacerbation, or lung or liver transplantation. Patients with CF were evaluated during periods of clinical stability (non-exacerbation periods). Healthy children were recruited via advertisements posted on faculty bulletin boards. The inclusion criterion for healthy children was being between 6 and 18 years old, and they were excluded if they had trouble understanding or following exercise test instructions.

### Patient and public involvement

Patients and the public were not involved in the design, conduct, reporting, or dissemination plans of this research.

### Procedure

Information about the diagnostic required for the study, cystic fibrosis genotype, height, weight, BMI z score (de Onis et al. 2007; Ogden et al. 2002), presence of impaired fasting glucose, medications used, the number of exacerbations during the previous year, Shwachman-Kulczycki score, the presence of chronic infection, P. aeruginosa, S. aureus, Burkholderia cepacia colonization, pancreatic insufficiency, and cystic fibrosis phenotype information (mild phenotype, severe phenotype, not yet classified) were recorded from the patient’s files. The Shwachman-Kulczycki score was calculated by pediatric pulmonologists experienced in CF. It was divided into four domains: general activity, physical examination, nutrition, and radiological findings, each with five possible sub-scores according to the degree of impairment. The scores of the four domains are summed to obtain the final score. Based on the scores, patients were categorized as excellent (86–100 points), good (71–85 points), average (56–70 points), poor (41–55 points), or severe (≤ 40 points) (Stollar et al. 2011).

### Pulmonary function

Forced expiratory volume in one second (FEV_1_), forced vital capacity (FVC), FEV_1_/FVC ratio, peak expiratory flow (PEF), and forced expiratory flow from 25% to 75% (FEF25–75%) were measured using a spirometer (Cosmed, Class II/Internally Powered Equipment, Italy) in accordance with the American Thoracic Society (ATS)/European Respiratory Society (ERS) standards (Miller et al. 2005). Parameters were expressed as a percentage of expected values (Knudson et al. 1976). Algorithms were employed to determine whether pulmonary function tests indicated obstructive, restrictive, or mixed pulmonary dysfunction (Johnson and Theurer 2014).

### Upper extremity functional exercise capacity

The 6-minute Pegboard and Ring (6PBRT) test assessed upper extremity exercise capacity using 12-hole boards with six holes in upper and lower rows 10 cm apart. Four wooden sticks, positioned based on shoulder width, allowed rings each 12 g to enter and exit. Twenty rings were used, and the test was repeated twice on the same day with 30-minute intervals (Celli et al. 1986). Heart rate (PE3000 Polar Electro, Finland), blood pressure, oxygen saturation (SpO_2_), breathing rate, dyspnea, and fatigue levels (Modified Borg scale) were immediately recorded before and after 6PBRT (Celli et al. 1986). The instructions were explained to the children. They were asked to wear the rings on both hands, first from the bottom up and then from the top down, for six minutes. Children were instructed not to interfere with the rings as they fell and to continue the test even if a ring dropped. They were allowed to rest and stop during the 6PBRT but were told to resume as soon as possible. Children were encouraged to use standard expressions during the test. Two rings that were removed and inserted at the same time were recorded as two separate rings. The highest 6PBRT score recorded from two attempts was used for analysis. There are no established reference values for the 6PBRT in the pediatric group. Results were compared with those of healthy controls.

### Muscle oxygenation

Muscle oxygenation was measured using the “Moxy^®^” monitor (Moxy, Fortiori Design LLC, Minnesota, USA) (Crum et al. 2017). The device was applied unilaterally to the dominant deltoid muscle during 6PBRT. After the previous evaluation protocol, the patient rested for 30 min, the device was placed, and the patient waited at least three minutes for the resting measurements and muscle oxygen saturation (SmO_2_) signal to stabilize. SmO_2_ and total hemoglobin (THb) levels were recorded at rest, during 6PBRT, and after one minute of recovery. Muscle oxygen saturation was expressed as a percentage (%), and THb as g/dl (Feldmann et al. 2019).

Upper extremity muscle oxygenation was assessed using a portable near-infrared spectroscopy device (Moxy^®^, Fortiori Design LLC, Minnesota, USA) (Crum et al. 2017). The sensor was positioned over the dominant deltoid muscle belly approximately at the midpoint between the lateral acromion and the deltoid tuberosity in accordance with established NIRS placement guidelines for upper-extremity musculature (Gorti et al. 2024). Before placement, the skin was cleaned with alcohol, and the probe was secured using an adhesive, light-occlusive covering to prevent ambient light intrusion and to minimize motion artefact during the dynamic arm activity of the 6PBRT (Stöggl et al., 2021).

All measurements were obtained in a standardized indoor environment with stable ambient temperature (22–24 °C). Participants were seated, and a minimum 3-minute stabilization period was allowed to ensure steady-state baseline values. Muscle oxygen saturation (SmO₂, %) and total hemoglobin concentration (THb, g/dL) were recorded continuously at 0.5 Hz during rest, throughout the entire 6PBRT (including all unsupported upper-extremity elevation phases), and during the first minute of post-test recovery. Raw signals were visually examined to identify and exclude segments affected by optical or motion-related artefacts (Jones et al. 2022; Yogev et al. 2023).

Resting, minimum, peak, and recovery values were calculated by averaging 5-second epochs corresponding to each physiological phase, consistent with previously published upper-extremity NIRS protocols. No additional filtering or normalization procedures were applied. Instead, relative changes (ΔSmO₂, ΔTHb) were used to account for inter-individual variability related to adiposity, optical path differences, and upper-extremity muscle morphology (Jones et al. 2022; Yogev et al. 2023). SmO₂ values are presented as percentages, and THb values are expressed in g/Dl (Feldmann et al. 2019).

### Peripheral muscle strength

Shoulder abductor and knee extensor isometric muscle strength were measured with a hand-held dynamometer (JTECH Power Track Commander, Baltimore, USA). Measurements were repeated three times on both sides. Children were properly positioned, and the relevant extremity areas were pressed against gravity until resistance broke. Children were warned not to change trunk position during the test. The highest value in Newtons (N) was used for analysis, with reference values for comparison. (Beenakker et al. 2001).

### Static balance

Static balance was evaluated using the “Biodex^®^ Biosway Portable Balance System” (Biodex^®^ Medical Systems Incorporated, Shirley, New York). Children were instructed to hold the black dot, representing their position, in the center of the innermost circle and maintain their balance for 20 s. A trial measurement was followed by three precise measurements with 10 s rest periods. The assessment was performed in four different stages, including eyes open and closed, as well as soft and hard floor conditions. Data collected from the system included the overall stability index, anterior-posterior (AP) stability index, and medial-lateral (ML) stability index, along with their standard deviations, which were recorded for analysis. The device measures individuals > 27 kg (Biodex Medical Systems Inc., 2014).

### Dynamic balance

Dynamic balance was assessed using the Y Balance Test. The directions of motion were named anterior, posteromedial, and posterolateral with reference to the stance foot. It was repeated three times on the right and left legs. The mean elongation distance was recorded in centimeters. The distance of each reach was divided by the child’s lower extremity length and then multiplied by 100, yielding a percentage result. The tests were repeated in cases where the child lost balance, moved the stance foot, or raised the heel (Shaffer et al. 2013).

### Statistical analyses

Statistical analysis was performed using the Windows-based SPSS 22 statistical analysis program. Sample size analysis (G*Power 3.0.10 system, Franz Faul, Universität Kiel, Germany) was performed using the pilot study data, with resting deltoid muscle oxygen saturation (10 participants each: 88.5 ± 8.02% versus 95 ± 2.36%) and a 95% power and 5% type 1 error probability. The study was designed to include at least 20 patients with CF and 20 healthy controls. Visual (histogram and probability plots) and analytical methods (Shapiro-Wilk test) were used to determine the normal distribution of the variables. Student t-test, Mann-Whitney U test, and Chi-square tests were used to compare normally distributed variables, non-normally distributed variables, and nominal data, respectively. Variables were descriptively expressed as mean, Standard Deviation (SD), mean difference, lower and upper limits of the 95% confidence interval (95%CI), median, interquartile range (IQR: 25–75%), U value, frequency (n), and percentage (%). The level of significance was set to *p* ≤ 0.05. Power analysis was performed to evaluate the statistical adequacy of the analyses and the effect size was calculated for the parameters 6PBRT score, SmO_2rest_ (%), SmO_2min_ (%), SmO_2max_ (%), ΔSmO_2_ (%), SmO_2average − min_ (%), SmO_2average − max_ (%), ΔSmO_2average_ (%), SmO_2recovery_ (%), SmO_2recovery − average_ (%), THb_min_ (g/dl), Thb_max,_ ΔTHb (g/dl), THb_recovery_ (g/dl), THb_rest_ (g/dl), quadriceps femoris, shoulder abductor muscle strength, the medial-lateral stability index.

## Results

**Fig. 1:**
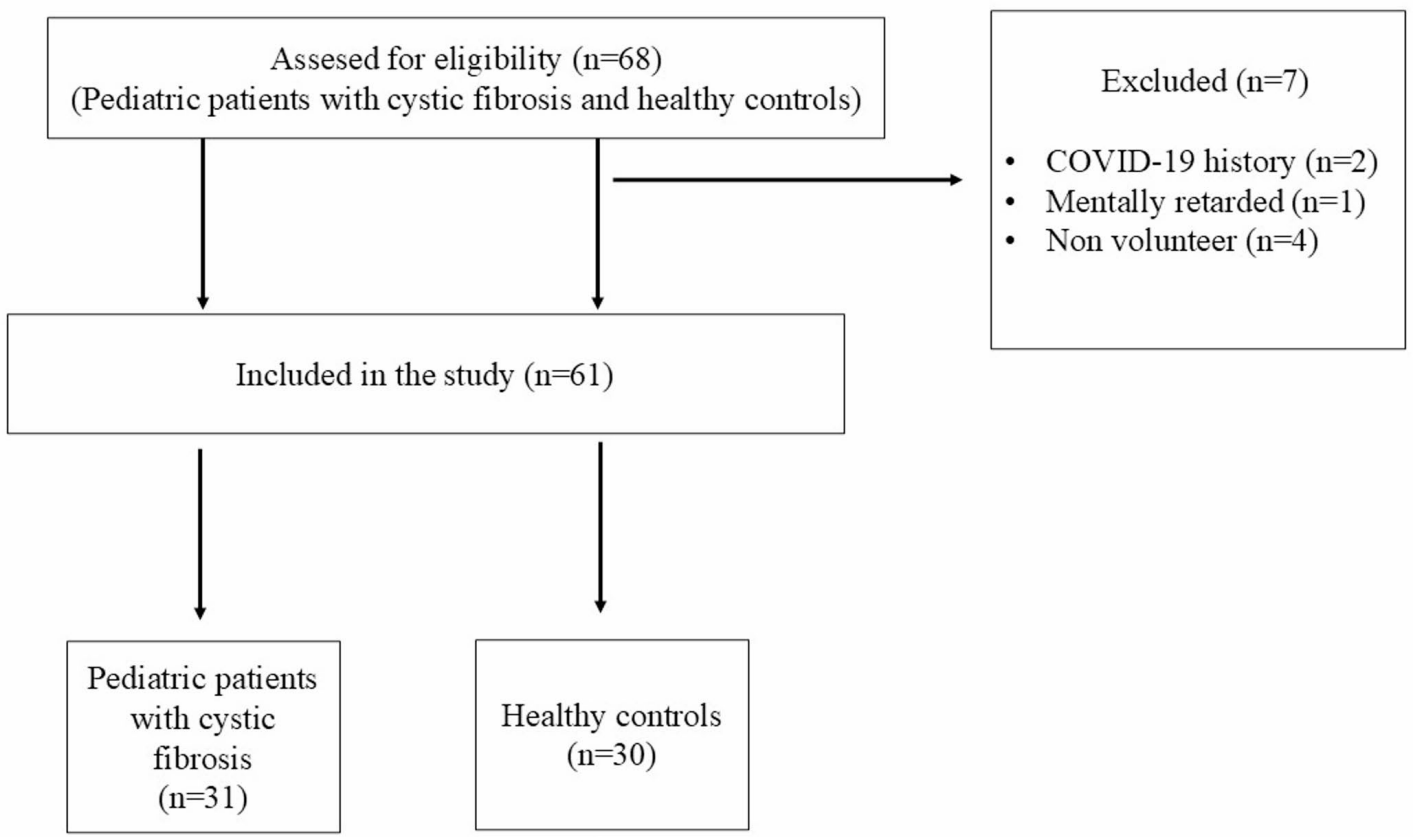
STROBE diagram of pediatric patients with cystic fibrosis and healthy controls

Thirty-seven pediatric patients with CF and 30 healthy controls were evaluated for eligibility. In this study, 31 of 37 pediatric patients with CF and 30 of 31 healthy controls were analyzed and compared (Fig. 1) The median diagnostic age was 4 (3–7) months, and the first symptoms of 21 pediatric patients (67.7%) started immediately after birth. None of the pediatric patients with cystic fibrosis were receiving highly effective CFTR modulator therapy (elexacaftor/tezacaftor/ivacaftor; ETI) during the study period.

**Fig. 2:**
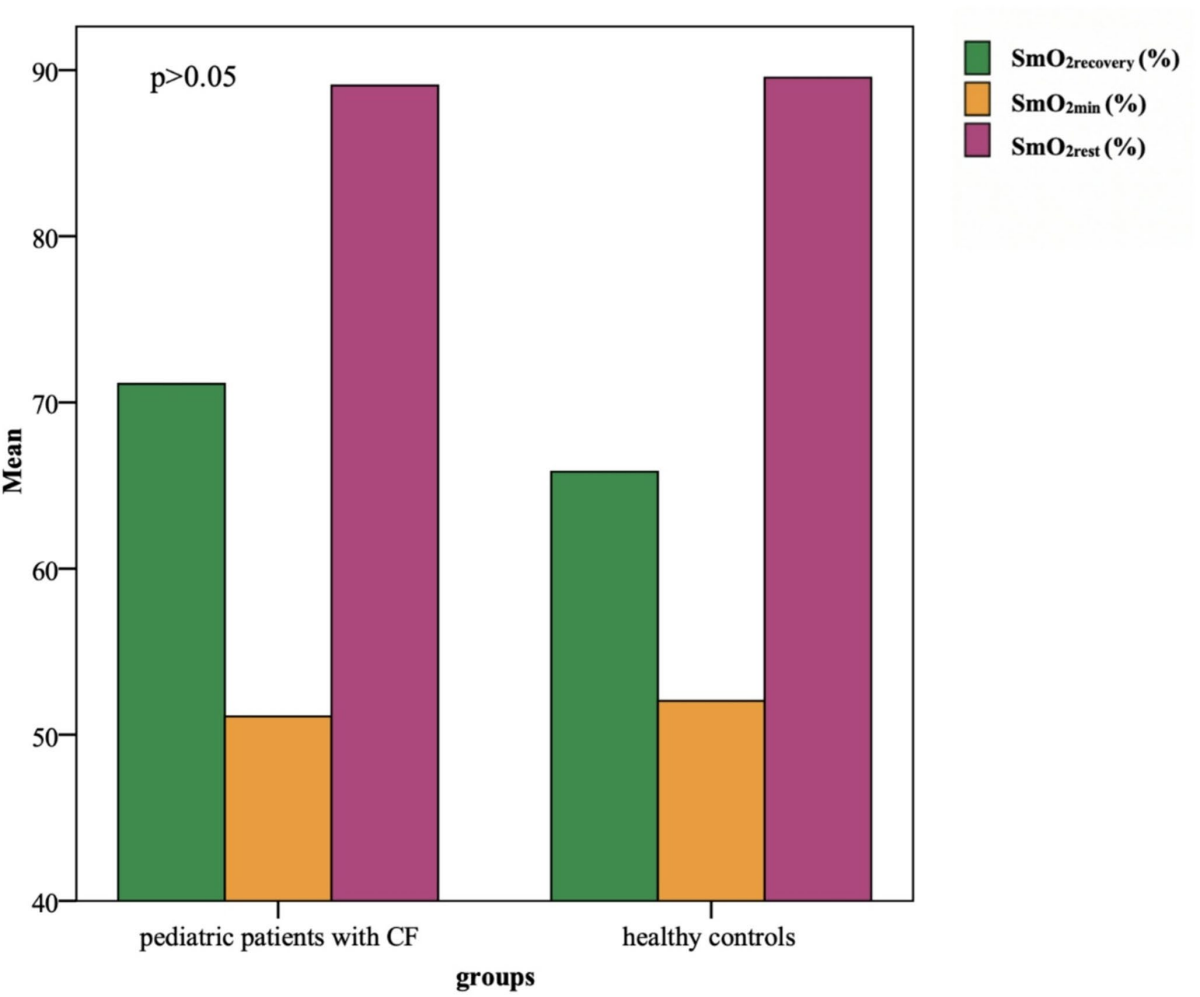
**(Online)** Deltoid muscle oxygen saturation (SmO₂) at rest, minimum SmO₂ during the 6-minute Pegboard and Ring Test, and recovery SmO₂ in pediatric patients with cystic fibrosis and healthy controls

The age, gender, and consanguineous marriages among parents were similar in groups (*p* > 0.05), and the patients’ z scores for weight, height, BMI, FEV_1_%, FVC%, PEF%, and FEF_25 − 75%_ were significantly decreased compared with healthy controls (*p* < 0.05, Table 1). FEV_1_/FVC, 6PBRT score (1 − β = 0.11, η^2^ = 0.72), Δ heart rate, Δ systolic and Δ diastolic blood pressure ΔSpO_2,_ Δ breathing frequency, Δ dyspnea, Δ general and Δ upper extremity fatigue, SmO_2rest_ (%) (1 − β = 0.05, η^2^ = 0.04), SmO_2min_ (%) (1 − β = 0.05, η^2^ = 0.06), SmO_2max_ (%) (1 − β = 0.05, η^2^ = 0.01), ΔSmO_2_ (%) (1 − β = 0.05, η^2^ = 0.05), SmO_2average − min_ (%) (1 − β = 0.05, η^2^ = 0.05), SmO_2average − max_ (%) (1 − β = 0.05, η^2^ = 0.01), ΔSmO_2average_ (%) (1 − β = 0.05, η^2^ = 0.06), SmO_2recovery_ (%) (1 − β = 0.05, η^2^ = 0.02), SmO_2recovery − average_ (%) (1 − β = 0.05, η^2^ = 0.03) (Fig. 2 (online)), THb_min_ (g/dl) (1 − β = 0.07, η^2^ = 0.12), Thb_max_ (g/dl) (1 − β = 0.05, η^2^ = 0.02), ΔTHb (g/dl) (1 − β = 0.06, η^2^ = 0.08), and THb_recovery_ (g/dl) (1 − β = 0.21, η^2^ = 0.30) were similar in groups (*p* > 0.05). THb_rest_ (g/dl) (1 − β = 0.47, η^2^ = 0.49) was higher in patients compared with controls (*p* < 0.05, Table 2). Quadriceps femoris (right; 1 − β = 0.41, η^2^ = 0.45; left; 1 − β = 0.58, η^2^ = 0.56), shoulder abductor muscle strength (right; 1 − β = 0.63, η^2^ = 0.59; left; 1 − β = 0.73, η^2^ = 0.67), and predicted values significantly decreased compared with healthy controls (*p* < 0.05, Table 2).

**Table 1:** Comparison of demographic and clinical characteristics in pediatric patients with CF and healthy controls

Characteristics	CF (*n* = 31)	Healthy controls (*n* = 30)	Mean difference %95CI/U	*p*
	X ± SS/ Median (IQR)	X ± SS/ Median (IQR)		
**Age**,** year**	10 (8–13)	10 (8–15)	455.5	0.89
**Male; female**,** n (%)**	19 (61.29%);12 (38.71%)	18 (60%);12 (40%)		0.918
**Weight**,** z score**	-0.78 ± 1.25	0.28 ± 0.98	-1.06 ((-1.64)-(-0.48))	**< 0.001** ^*^
**Height**,** z score**	-0.40 ± 1.17	0.47 ± 1.17	-0.86 ((-1.44)-(-0.29))	0.004*
**BMI**,** z score**	-0.83 ± 1.57	-0.10 ± 1.39	-0.73 (-1.49-0.02)	0.059*
**Malnutrition**,** n (%)**	6 (19.35%)	0 (0%)		**NA**
**FEV**_**1**_, **%**	79.48 ± 25.8	104.07 ± 17.59	-24.58 ((-35.93)-(-13.23))	**< 0.001** ^*^
**FVC**,** %**	78.91 ± 22.4	99.47 ± 16.08	-20.55 ((-30.57)-(-10.53))	**< 0.001** ^*^
**FEV**_**1**_ **/FVC**	86.93 ± 10.91	89.63 ± 6.47	-2.7 (-7.3-1.9)	0.245
**PEF**,** %**	79.49 ± 24.49	92.63 ± 16.88	-13.14 ((-23.96)-(-2.33))	0.018*
**FEF**_**25 − 75**,_ **%**	79.7 ± 39.34	113.6 ± 28.12	-33.9 ((-51.47)-(-16.33))	**< 0.001** ^*^
**Pulmonary exacerbation (last year)**	1 (1–1,5)			
**Cystic fibrosis-associated diabetes**,** n (%)**	3 (9.67%)			
**Impaired fasting glucose**,** n (%)**	6 (19.35%)			
**Pancreatic insufficiency**,** n (%)**	27 (87.09%)			
**Culture of sputum**				
Pseudomonas aeruginosa, n (%)	3 (9.67%)			
Staphylococcus aureus, n (%)	8 (25.8%)			
E. coli, n (%)	1 (3.22%)			
**Sweat chloride concentrations (mmol/L)**	104.74 ± 28.01			
**Shwachman-Kulczycki score (points)**	85 (80–95)			
Excellent (86–100), n (%)	15 (48.4%)			
Good (71–85), n (%)	9 (29%)			
Mild (56–70), n (%)	6 (19.4%)			
Moderate (41–55), n (%)	-			
Severe < 40, n (%)	1 (3.2%)			
**Drug use**				
Dornase alpha	31 (100%)			
Tobramycin	1 (3.22%)			
Hypertonic saline	13 (41.93%)			
Salbutamol	12 (38.7%)			
Proton pump inhibitor	29 (93.54%)			
Pancreatic enzyme replacement therapy	29 (93.54%)			
Insulin	2 (6.45%)			
Use of additional vitamins and minerals	29 (93.54%)			
Use of additional salt	10 (32.25%)			
**Phenotype classification**				
Mild phenotype	9 (29.03%)			
Severe phenotype	13 (41.93%)			
Not classified	9 (29.03%)			
**Pulmonary rehabilitation modalities** **Using airway clearance techniques**	31 (100%)			

Descriptive analyses were presented using (X ± SD) and median (IQR) for normally and non-normally distributed variables, respectively.

Student’s t-test *p *<* 0.05^#^

* BMI* body mass index,* FEV*_1_ forced expiratory volume in 1 s,* FVC* forced vital capacity,* PEF* peak expiratory flow,* FEF*_25–75_%, forced expiratory flow 25–75%,* CI* confidence interval

**Table 2:** Comparison of 6PBRT scores, end-test difference values, deltoid muscle oxygenation, total hemoglobin levels, and peripheral muscle strength values in pediatric patients with CF and healthy controls

	CF (*n* = 31)	Healthy controls (*n* = 30)	Mean difference %95CI/U	*p*
	X ± SS/Median (IQR)	X ± SS/Median (IQR)		
6PBRT, n	305.84 ± 88.66	290.13 ± 79.52	15.7 (-27.49-58.9)	0.470
ΔHR, beat/min	9.26 ± 7.48	12.4 ± 8.89	-3.14 (-7.34-1.06)	0.14
ΔSBP, mmHg	5 (0–10)	4 (0–10)	430	0.602
ΔDBP, mmHg	0 (0–10)	4,5 (0–10)	436	0.659
ΔSpO_2_, %	0 (-1-1)	0 (-1-0)	416	0.461
ΔBreathing frequency, breath/min	4 (4–8)	4 (0–8)	375	0.168
ΔDyspnea, (MBS) (0–10)	0 (0–1)	0 (0–0)	347	0.056
ΔGeneral fatigue, (MBS) (0–10)	1 (0–3)	0 (0–2)	375	0.177
ΔUpper extremity fatigue, (MBS) (0–10)	3 (2–5)	3 (2–4)	369.5	0.161
SmO_2rest,_ %	93 (86–94)	93 (79–94)	442	0.738
SmO_2 min_, %	52.32 ± 15.95	53.5 ± 18.55	-1.17 (-10.03-7.67)	0.791
SmO_2max,_ %	72.22 ± 11.59	73.49 ± 16.11	-1.27 (-8.44-5.89)	0.724
ΔSmO_2,_ %	17 (12–22)	20 (13–25)	409	0.419
SmO_2average − min,_ %	54.32 (43–61)	55.66 (46–63)	445	0.773
SmO_2average − max,_ %	70.75 ± 11.63	71.94 ± 16.75	—1.19 (-8.55-6.17)	0.747
ΔSmO_2average,_ %	14 (10–19)	16.5 (12–23)	397.5	0.30
SmO_2recovery,_ %	71.39 ± 13.78	66.84 ± 17.71	4.55 (-3.56-12.67)	0.266
SmO_2recovery − average,_ %	69.77 ± 13.68	66.1 ± 17.47	3.67 (-4.35-11.69)	0.363
THb_rest,_ g/dl	12.34 ± 0.65	12.07 ± 0.42	0.27 (0-0.55)	0.056
THb_min,_ g/dl	11.84 (11.59–12.12)	11.67 (11.47–12.19)	419	0.507
Thb_max,_ g/dl	12.26 (11.9-12.57)	12.31 (11.85–12.85)	437	0.686
ΔTHb, g/dl	0.22 (0.15–0.44)	0.31 (0.16–0.47)	400	0.348
THb_recovery,_ g/dl	12 (11.69–12.35)	11.94 (11.54–12.4)	443	0.751
Quadriceps femoris muscle strength (left), N	134 (110–211)	180 (156–382)	274.5	**0.006** ^**#**^
Quadriceps femoris muscle strength (left), %	63.58 (54.04–78.85)	98.2 (70.06-114.65)	208	**< 0.001** ^**#**^
Shoulder abductors muscle strength (left), N	92.4 (70–118)	119.5 (88–169)	299	**0.017** ^**#**^
Shoulder abductors muscle strength (left),%	72.46 (58.14–91.91)	91.21 (81.3-111.36)	245	**0.002** ^**#**^

Descriptive analyses were presented using (X ± SD) and median (IQR) for normally and non-normally distributed variables, respectively

Mann–Whitney U test ^#^*p* < 0.05

*6PBRT* 6-min Pegboard and ring test,* c* count, %, percentage; Δ, the difference between post and pretest,* HR* heart rate,* SBP* systolic blood pressure,* DBP* diastolic blood pressure,* SpO*_2_ oxygen saturation,* MBS* modified Borg scale,* SmO*_2_ muscle oxygen saturation, *SmO*_2res**t**_, muscle oxygen saturation at rest,* SmO*_2min_, minimum muscle oxygen saturation during the test,* SmO*_2max_, maximum muscle oxygen saturation during the test; *SmO*_2average − min_, minimum mean muscle oxygen saturation during the test; *SmO*_2average − max_, maximum mean muscle oxygen saturation during the test,* SmO*_2recovery_, recovery muscle oxygen saturation,* SmO*_2recovery − average_, recovery mean muscle oxygen saturation,* THb* total hemoglobin level,* TH*_rest_, resting total hemoglobin level,* THb*_min_, minimum total hemoglobin level during test,* Thb*_max_, maximum total hemoglobin level during test,* THb*_recovery_, recovery total hemoglobin level,* N*, newton,* KgF* kilogram-force,* CI* confidence interval

**Table 3:** Comparison of static and dynamic postural stability in pediatric patients with CF and healthy controls

	CF (*n* = 19)	Healthy controls (*n* = 20)	Mean difference %95CI/U	*p*
	X ± SS/Median (IQR)	X ± SS/Median (IQR)		
Static postural stability				
**Eyes open and hard ground**				
• General stability index, %	0.8 (0.6–1.3)	1.1 (0.8–1.2)	182.5	0.643
• AP stability index, %	0.7 (0.4–1.1)	0.7 (0.6-1)	198.5	0.978
• ML stability index, %	0.4 (0.2–0.5)	0.4 (0.2–0.7)	181	0.612
• Overall sway index, %	0.52 (0.39–0.93)	0.59 (0.44–0.82)	385	0.903
• AP sway index, %	0.54 (0.39–1.01)	0.57 (0.42–0.79)	193	0.860
• ML sway index, %	0.34 (0.26–0.44)	0.35 (0.19–0.51)	189	0.776
**Eyes open and soft ground**				
• General stability index, %	1.8 (1.2–2.5)	1.4 (1-1.95)	129	0.086
• AP stability index, %	1.1 (0.9–1.8)	1 (0.75–1.55)	157	0.352
• ML stability index, %	1 (0.6–1.2)	0.7 (0.45–0.95)	111.5	**0.027** ^**#**^
• Overall sway index, %	1.02 ± 0.39	1.01 ± 0.73	0 (-0.37-0.38)	0.973
• AP sway index, %	0.84 (0.74–1.43)	0.78 (0.6–1.05)	142.5	0.182
• ML sway index, %	0.73 ± 0.27	0.58 ± 0.24	0.15 (-0.01-0.31)	0.077
**Eyes close and hard ground**				
• General stability index, %	1.4 (1.1–1.6)	1.2 (0.9-2)	186	0.714
• AP stability index, %	1 (0.8–1.3)	1 (0.7–1.5)	198	0.967
• ML stability index, %	0.6 (0.4-1)	0.5 (0.3–0.9)	157.5	0.253
• Overall sway index, %	0.76 (0.6-1)	0.73 (0.51–1.06)	194.5	0.892
• AP sway index, %	0.81 (0.6-1)	0.75 (0.53–1.13)	187	0.735
• ML sway index, %	0.44 (0.36–0.7)	0.37 (0.21–0.57)	159.5	0.278
**Eyes close and soft ground**				
• General stability index, %	2.3 (2-3.5)	2.15 (1.8–3.45)	169	0.555
• AP stability index, %	1.8 (1.5–2.4)	1.75 (1.3–2.45)	178	0.736
• ML stability index, %	1 (0.9–1.6)	1.05 (0.9–1.75)	171	0.591
• Overall sway index, %	1.36 (1.16–1.98)	1.22 (1.04–1.73)	165	0.482
• AP sway index, %	1.44 (1.13–1.82)	1.3 (1.04–1.7)	171	0.593
• ML sway index, %	0.87 (0.73–1.33)	0.86 (0.71–1.26)	171.5	0.603
**Dynamic postural stability**				
**Right lower extremity**				
• Anterior	1.05 ± 0.20	0.97 ± 0.13	0.07 (0-0.16)	0.071
• Posteromedial	0.84 (0.67–0.96)	0.81 (0.74–0.94)	432	0.634
• Posterolateral	1.05 ± 0.21	0.97 ± 0.15	0.08 (-0.01-0.17)	0.087
**Left lower extremity**				
• Anterior	1.05 ± 0.19	0.93 ± 0.13	0.12 (0.03–0.2)	0.005*
• Posteromedial	0.85 ± 0.18	0.83 ± 0.15	0.02 (-0.06-0.11)	0.562
• Posterolateral	1.07 (0.95–1.15)	0.98 (0.9–1.09)	333.5	**0.058** ^#^

Descriptive analyses were presented using (X ± SD) and median (IQR) for normally and non-normally distributed variables, respectively.

Student’s t-test *p < 0.05, Mann–Whitney U test ^#^*p* < 0.05.

*AP* anterior-posterior,* ML* medial-lateral,* cm* centimeter

Since the “Biodex Biosway ^®^ Portable Balance System” system measured individuals > 27 kg, data from 19 patients with CF and 20 healthy children were presented. The medial-lateral stability index (1 − β = 0.83, η^2^ = 0.76) of pediatric patients with CF on soft ground with eyes open was statistically significantly higher (p < 0.05). Static postural stability indices on soft ground with eyes open and closed, general stability index on soft ground with eyes open, anterior-posterior stability index, and sway indices of the groups were similar (p > 0.05, Table 3). Right lower extremity anterior, posteromedial, and posterolateral measurement values and left lower extremity posteromedial and posterolateral measurement values of the groups were similar (p > 0.05, Table 3). Pediatric patients’ left lower extremity anterior and posterolateral measurement values were statistically significantly higher than healthy controls (p < 0.05, Table 3).

## Discussion

In this study, a hypothesis was created assuming that upper extremity exercise capacity, muscle oxygen metabolism, peripheral muscle strength, and static and dynamic balance were impaired in pediatric patients with CF. However, the findings obtained supported some results of the hypothesis such as decreased lower and upper extremity muscle strength, impaired pulmonary functions; however, it was shown that upper extremity exercise capacity, muscle oxygen saturation, total hemoglobin level, and static and dynamic balance parameters were preserved in patients with relatively good and excellent clinical conditions (77.41%, Shwachman Kulczycki). It should be noted that this study particularly highlights the results of pediatric patients with CF despite good clinical conditions, as patients with good clinical conditions constitute the majority in the management of new treatment modalities.

Although the sample size calculation at the beginning of this study was based on the deltoid muscle oxygenation parameter, it was observed at the end of the study that the statistical power of balance parameters was higher. The significant relationships between upper extremity exercise capacity and balance revealed (Riach and Hayes 1987) the clinical and conceptual importance of evaluating these two components together.

In this study, upper extremity exercise capacity was evaluated with 6PBRT (Celli et al. 1986), a safe and valuable tool in healthy adolescents (Leite et al. 2021). No adverse events were reported. This is the first study to assess upper extremity exercise capacity in CF patients, showing preserved capacity despite impaired pulmonary function and muscle weakness. Compared with Leite et al. (Leite et al. 2021), the 6PBRT score was lower, though capacity was maintained. Previous studies in confirmed 6PBRT as valid and reliable (Calik-Kutukcu et al. 2022), with younger age, better clinical status, pulmonary function, and muscle strength associated with higher performance (Calik-Kutukcu et al. 2022). Preservation of muscle oxygen metabolism may explain the maintained capacity in our cohort. While mild to moderate CF is linked to reduced O₂ utilization (Rodriguez-Miguelez et al. 2021), reference values for pediatric 6PBRT remain limited.

A central finding of the present study is the preservation of deltoid muscle oxygenation despite the presence of peripheral muscle weakness and lower BMI z-scores in pediatric patients with CF. Although this finding may initially appear paradoxical, current evidence indicates that reductions in muscle strength and nutritional status do not necessarily coincide with early impairments in local muscle oxygen delivery or utilization, particularly during low-to-moderate intensity functional exercise. In CF, peripheral muscle weakness is predominantly related to reduced muscle mass, altered neuromuscular activation, and diminished force-generating capacity rather than to early microvascular dysfunction (Troosters et al. 2010; Radtke et al. 2019). Accordingly, muscle weakness and preserved muscle oxygenation may coexist, especially in patients with mild disease severity.

In the present cohort, most patients demonstrated relatively preserved pulmonary function and a stable clinical status, supported by regular airway clearance therapy. This clinical profile likely facilitated adequate systemic oxygen transport and limited ventilatory constraints during exercise. Previous studies have shown that patients with less advanced pulmonary impairment are able to maintain effective matching between oxygen delivery and oxygen utilization during submaximal exercise (Werkman et al. 2016; Saynor et al. 2020). Thus, the preserved upper extremity muscle oxygenation observed in this study likely reflects sufficient cardiopulmonary support rather than the absence of underlying muscular involvement.

Although BMI Z scores were lower and a proportion of patients met criteria for malnutrition, BMI alone does not fully reflect skeletal muscle oxidative capacity or microvascular function. Longitudinal evidence suggests that early nutritional compromise in CF primarily affects growth and muscle strength, whereas alterations in muscle oxygenation and oxidative metabolism tend to emerge later, in association with disease progression, worsening pulmonary function, or increased systemic inflammation (Zemel et al. 2000; Coelho et al. 2020). In addition, skeletal muscle fiber composition may contribute to these findings. Pediatric patients with mild CF appear to retain a relatively higher proportion of oxidative (type I) muscle fibers and mitochondrial capacity, supporting efficient oxygen utilization and delaying local muscle deoxygenation during functional tasks (Troosters et al. 2010; Radtke et al. 2019).

Exercise modality is another important consideration. Upper extremity functional tasks, such as the 6PBRT, recruit a smaller muscle mass and impose lower absolute metabolic and ventilatory demands compared with lower limb exercise. This reduced physiological demand may allow adequate oxygen delivery even in the presence of peripheral muscle weakness or nutritional compromise. In contrast, impairments in muscle oxygenation have been reported more consistently during lower extremity exercise or in adult patients with greater disease burden (Coelho et al. 2020; Rodriguez-Miguelez et al., 2021). Collectively, these findings suggest that peripheral muscle weakness and nutritional impairment may precede detectable abnormalities in muscle oxygenation, particularly in the early stages of disease.

Importantly, the interpretation of muscle oxygenation data in CF is currently limited by the absence of disease-specific reference values and clinically meaningful cut-off points. At present, there are no established thresholds to define impaired muscle oxygenation in pediatric CF populations. Therefore, future studies should aim to establish CF specific cut-off values for muscle oxygenation parameters, investigate their relationship with disease severity, nutritional status, muscle strength, and pulmonary function, and integrate complementary physiological markers such as muscle morphology, fiber type distribution, inflammatory indices, and hemoglobin concentration. Such work may improve the clinical interpretability of muscle oxygenation measurements and support their integration into comprehensive assessment and rehabilitation strategies in CF.

The present study showed weakness in quadriceps femoris and shoulder abductors in pediatric CF patients. Previous studies also reported reduced peripheral muscle strength (Arikan et al., 2015; Pinet et al. 2003), while others found it preserved (Hussey et al. 2002). Similar to our findings, Arikan et al. (Arikan et al.,^2015^) and Pinet et al. (Pinet et al. 2003) reported weakness in shoulder abductors and knee extensors, whereas Hussey et al. (Hussey et al. 2002) found preserved knee extensor strength. In CF, impaired respiratory function, low lean body mass, oxidative stress, inactivity, corticosteroid use, and reduced exercise capacity contribute to muscle weakness (Hussey et al. 2002; Sharma et al. 2001).

The borderline between-group difference in BMI Z scores is also clinically relevant for interpreting peripheral muscle strength outcomes. In pediatric CF, peripheral muscle weakness is a well-described feature and has been linked to reductions in fat-free mass and nutritional depletion, which can limit force generation capacity and contribute to lower exercise tolerance (de Meer et al. 1999). In addition, longitudinal registry data indicate that growth and nutritional status indices are not stable in children with CF and are associated with clinically meaningful changes in health status over time (Zemel et al. 2000). Accordingly, the lower peripheral muscle strength observed in the CF group in the present study is biologically consistent with expected differences in nutritional status in CF, and it is plausible that even borderline differences in BMI z-scores may reflect underlying differences in lean mass and muscle function. This interpretation aligns with the broader CF exercise physiology literature emphasizing skeletal muscle dysfunction and nutritional depletion as key contributors to impaired physical performance in CF (Radtke et al. 2019).

Most studies addressed adolescents and adults (Hussey et al. 2002; Pinet et al. 2003) while we evaluated younger children. Early weakness in patients aged 10 (8–13) years, with malnutrition (19.35%) and relatively preserved lung volumes, underlines the importance of integrating cardiopulmonary rehabilitation early in treatment.

Upper extremity exercise capacity is closely related to balance and postural control in CF. Arm activities enhance trunk stabilization, supporting postural mechanisms (Riach and Hayes 1987) Weakness in trunk and upper extremity muscles, common in CF, may impair limb function and balance (Burtin et al. 2013). Literature indicates that upper extremity exercises improve trunk stability and balance in both sitting and standing (Lee and Choi 2017). Therefore, evaluating exercise capacity together with balance provided conceptual integrity in this study.

In the present study, static balance assessment using the Biodex Biosway^®^ Portable Balance System was not feasible in children with a body weight below 27 kg due to device-related limitations. Therefore, children from both the CF group and the healthy control group who weighed less than 27 kg were excluded from the Biodex-based balance assessment to avoid invalid or unreliable measurements. Importantly, this technical exclusion criterion was applied equally to both groups, and participants excluded due to low body weight were present in both cohorts. Although this resulted in a reduced sample size for the Biodex analysis, comparisons of basic demographic and clinical parameters, including age, BMI z-scores, and pulmonary function, revealed no statistically significant differences between participants who were included and those who were excluded, supporting the representativeness of the analyzed subgroup. Nevertheless, it should be acknowledged that this limitation may have led to an underrepresentation of more frail or undernourished children with CF, potentially resulting in an underestimation of balance impairments when assessed using platform-based systems. To address this limitation and minimize the risk of selection bias, dynamic functional balance was additionally assessed using the Y-Balance Test, a validated and widely used functional balance test in pediatric populations (Plisky et al. 2006; Shaffer et al. 2013). Unlike the Biodex Biosway^®^ Portable Balance System, the Y-Balance Test does not impose a minimum body weight requirement and was successfully administered to all children in both groups, allowing for a broader and more inclusive evaluation of balance capacity.

Although this is a common limitation in pediatric studies, underweight or malnourished children with CF are known to be at increased risk for balance impairments due to decreased muscle mass, impaired neuromuscular control, and lower physical activity levels (Bell et al. 2020; Nicolson et al. 2022). Malnutrition in CF is associated with muscle weakness and postural instability, which may negatively affect daily functional activities and increase the risk of falls Bell et al. 2020; Nicolson et al. 2022). Therefore, assessing balance capacity even in children with low body weight is of clinical importance. The inclusion of the Y-Balance Test enabled a comprehensive evaluation of dynamic balance in all CF participants, including those with underweight or malnutrition, and increased both the clinical relevance and methodological robustness of balance assessment in this study (Plisky et al. 2006; Shaffer et al. 2013).

In the present study, patients with CF exhibited impaired medial–lateral stability under static conditions, while simultaneously demonstrating greater anterior and posterolateral reach distances on the Y-Balance Test compared with healthy controls. Although longer reach distances in dynamic balance tests are commonly interpreted as superior balance performance, accumulating evidence suggests that such findings may also reflect compensatory motor strategies rather than true preservation of postural control. In individuals with chronic disease, balance performance is often maintained through adaptive mechanisms that compensate for underlying neuromuscular deficits instead of full recovery of postural stability (Horlings et al. 2008).

Peripheral muscle weakness is a well recognized feature in CF and has been shown to be associated with impaired postural control and balance performance (de Meer et al. 1999; Lima et al. 2014). In particular, reductions in peripheral muscle strength may compromise ankle-based postural strategies, leading individuals to rely more heavily on proximal musculature, trunk stabilization, and stance limb control to maintain functional balance. Such proximal-dominant compensatory strategies have been described as a common adaptive response when frontal-plane stability is impaired, especially in the presence of neuromuscular or proprioceptive deficits (Horlings et al. 2008; Sozzi 2022).

Accordingly, the higher anterior and posterolateral reach distances observed in the CF group in the present study may represent an adaptive compensatory strategy driven by peripheral muscle weakness rather than unequivocal superiority of dynamic balance. The coexistence of impaired ML stability and preserved or enhanced dynamic reach performance suggests a dissociation between static postural control and functional balance capacity. The Y-Balance Test, which challenges multi-directional reach under single-limb support, may therefore capture functional adaptations that allow children with CF to maintain dynamic balance during daily activities, while masking subtle balance deficits that become evident only under static or sensory-challenged conditions. Further studies integrating muscle strength assessments, kinematic analyses, and neuromuscular evaluations are warranted to clarify the relative contributions of peripheral muscle weakness and compensatory strategies to balance performance in pediatric CF populations.

Medial-lateral stability, crucial for movements like changing direction and stair climbing, affects fall risk and mobility in CF patients. Its decline can impair gait and posture, increase fear of falling, and reduce participation in physical activity in patients with CF and further deteriorate musculoskeletal health by reinforcing a sedentary lifestyle (Horak 2006). In addition, the deterioration of balance in the frontal plane can cause psychosocial effects by increasing the fear of falling and negatively affect the individual’s quality of life.

It should be noted that the deterioration in the medial-lateral stability index detected in patients with CF is not only a biomechanical but also a clinical symptom impacting daily functioning.

As discussed above, balance performance in children with CF should be interpreted within a broader physiological and methodological context. Although BMI Z scores differed between groups at a borderline level, static balance assessment was necessarily restricted to children meeting the minimum body weight requirement, which may have limited the extent to which nutritional differences were reflected in platform-based outcomes. In contrast, dynamic balance assessment included all participants, allowing potential disease-related and anthropometric influences to become more apparent at the functional level.

Importantly, lower BMI Z scores and nutritional compromise are well-established features of CF and have been linked to reduced peripheral muscle mass and strength, which may influence postural control and balance performance (de Meer et al. 1999; Zemel et al. 2000). In this context, the dynamic balance findings observed in the present study may reflect the combined influence of disease-related peripheral muscle weakness and nutritional status, acting alongside the adaptive motor strategies discussed above. Thus, even when balance performance appears preserved at the task level, it may still be shaped by underlying neuromuscular and anthropometric constraints inherent to CF (Lima et al. 2014).

There are emerging studies examining static and dynamic balance (postural stability) in patients with CF (Dik et al. 2020; Kenis-Coskun et al. 2017; Lima et al. 2014). Besides computer-based systems, which are more reliable and objective, clinical tests are commonly used in these studies to assess balance (Dik et al. 2020; Kenis-Coskun et al. 2017; Lima et al. 2014). Two studies have shown that anterior-posterior, medial static balance (Zeren et al. 2019), and dynamic balance are affected (Dik et al. 2020), whereas a study using reliable methods found static balance to be preserved in patients with CF (Kenis-Coskun et al. 2017). The literature suggests that peripheral and inspiratory muscle weakness, systemic inflammation, nutritional status, and the use of aminoglycoside antibiotics for pulmonary exacerbations may contribute to postural instability in patients with CF (Beauchamp et al. 2012; Janssens et al. 2013; Smith et al. 2016; Tudorache et al. 2015). Additionally, one study indicated that peripheral muscle weakness leads to impaired balance in the medial-lateral range (Lima et al. 2014). In our current study, many patients had peripheral muscle weakness, which could explain the impaired balance in the medial-lateral range with eyes open. In contrast, Lima et al. (Lima et al. 2014), who used a computer-based system in patients with CF, reported poor static balance with eyes closed. In our study, static balance with eyes closed was maintained, likely due to better integration of somatosensory and vestibular inputs, supporting adequate postural control (Mainenti et al. 2011). Lima et al. also noted (Lima et al. 2014) a higher number of patients with CF-related diabetes compared to our current sample. It is known that postural stability is affected in patients with type 2 diabetes (Silva et al. 2019).

In contrast to the current study, Dik et al. (Dik et al. 2020) used the functional reach test to show poor dynamic balance in patients with CF. They suggested that this could be related to the aminoglycoside antibiotics, commonly used during pulmonary exacerbations, which cause vestibulotoxicity by affecting the vestibular system (Dik et al. 2020). Vestibulotoxicity was not examined in this study, but the number of patients who experienced pulmonary exacerbations in the past year was small. Furthermore, the variety of test methods used to assess dynamic balance may have contributed to the different results. More studies are needed to explore the effects of static and dynamic postural stability in patients with cystic fibrosis-related diabetes and frequent pulmonary exacerbations.

### Limitations

The primary limitation of this study is that data collection occurred during the COVID-19 pandemic, which may have influenced parameters such as exercise capacity, muscle oxygenation, and balance in healthy children. A secondary limitation of the present study is that static balance assessment using the Biodex Biosway^®^ Portable Balance System was not feasible in children with a body weight below 27 kg due to device-related constraints. Consequently, more frail or undernourished children with cystic fibrosis may have been underrepresented in the platform-based static balance analysis, potentially leading to an underestimation of balance impairments in this population. Although this exclusion criterion was applied equally to both the cystic fibrosis and healthy control groups, and no statistically significant differences were observed between included and excluded participants in terms of basic demographic and clinical characteristics, this limitation should be considered when interpreting the static balance findings. To mitigate this limitation and reduce the risk of selection bias, dynamic functional balance was additionally assessed using the Y-Balance Test, which can be administered without body weight restrictions and was successfully applied to all participants, thereby enhancing the inclusiveness, clinical relevance, and methodological robustness of the balance assessment. In the present study, the impact of upper extremity functional exercise capacity on balance was examined using standardized static and dynamic balance assessments. Given the holistic nature of postural control, these balance tests were expected to reflect overall balance performance. However, balance assessments performed in body-centered or sitting positions (e.g., the Functional Reach Test or Sitting Balance Test) were not included, which may have limited the ability to directly isolate the contribution of upper extremity performance to balance control.

## Conclusion and future directions

In pediatric patients with CF and relatively mild disease impairment, upper extremity exercise capacity, deltoid muscle oxygenation, and balance are preserved, whereas peripheral muscle strength is already reduced. This dissociation suggests that early peripheral muscular impairment may precede detectable limitations in functional exercise performance. These findings provide a clinically relevant framework indicating that assessments of exercise capacity and muscle oxygen metabolism should not be restricted to patients with advanced disease severity. Instead, future research should systematically investigate how progressive pulmonary dysfunction influences upper extremity exercise capacity and peripheral muscle oxygenation across the disease spectrum, particularly in patients with more severe cystic fibrosis. In addition, although peripheral muscle strength was evaluated, trunk (core) muscle strength was not assessed. Considering the critical role of trunk musculature in upper extremity function and postural stability, future studies incorporating core muscle strength measurements and body-centered balance assessments may provide a more comprehensive understanding of the interactions between upper extremity performance, balance, and neuromuscular control in pediatric patients with CF. From a clinical perspective, our results support the early implementation of comprehensive pulmonary rehabilitation programs that incorporate both upper and lower extremity aerobic training, aiming not only to preserve functional performance but also to address early peripheral muscle weakness in pediatric CF.
